# Fabrication and Characterization of a Micro Methanol Sensor Using the CMOS-MEMS Technique

**DOI:** 10.3390/s151027047

**Published:** 2015-10-23

**Authors:** Chien-Fu Fong, Ching-Liang Dai, Chyan-Chyi Wu

**Affiliations:** 1 Department of Mechanical Engineering, National Chung Hsing University, Taichung 402, Taiwan; E-Mail: fongjeff@livemail.tw; 2 Department of Mechanical and Electro-Mechanical Engineering, Tamkang University, Tamsui 251, Taiwan; E-Mail: ccwu@mail.tku.edu.tw

**Keywords:** methanol sensor, tin dioxide, cadmium sulfide, heater

## Abstract

A methanol microsensor integrated with a micro heater manufactured using the complementary metal oxide semiconductor (CMOS)-microelectromechanical system (MEMS) technique was presented. The sensor has a capability of detecting low concentration methanol gas. Structure of the sensor is composed of interdigitated electrodes, a sensitive film and a heater. The heater located under the interdigitated electrodes is utilized to provide a working temperature to the sensitive film. The sensitive film prepared by the sol-gel method is tin dioxide doped cadmium sulfide, which is deposited on the interdigitated electrodes. To obtain the suspended structure and deposit the sensitive film, the sensor needs a post-CMOS process to etch the sacrificial silicon dioxide layer and silicon substrate. The methanol senor is a resistive type. A readout circuit converts the resistance variation of the sensor into the output voltage. The experimental results show that the methanol sensor has a sensitivity of 0.18 V/ppm.

## 1. Introduction

Methanol sensors can be applied in electronic noses [1,2], fuel cells [3], disease diagnosis [4] and environment Monitoring [5]. Various microsensors [6,7] have been developed using micromachining technology. This technology was used to miniaturize traditional gas sensors as gas microsensors [8,9,10]. Gas microsensors have the advantages of small size, low noise, high sensitivity, low cost and easy mass-production [11,12]. Many methanol microsensors have been fabricated using micromachining technology. For instance, Tang *et al.* [13] proposed a micro methanol sensor fabricated using MEMS technology. The sensitive material of the sensor was SnO_2_-ZnO composite nanofibers prepared by the stepwise-heating electrospinning method. A Si/SiO_2_/Ti/Pt substrate was fabricated by thermal oxidation, photolithography, sputtering and liftoff. The sensitive SnO_2_-ZnO material was deposited on the Si/SiO_2_/Ti/Pt substrate. Guo *et al*. [14] used MEMS technology to manufacture an alcohol vapor sensor. The sensitive material of the sensor was epoxy acrylate polymer. The sensor consisted of a Si microbridge embedded with piezoresistive Wheatstone circuit and an epoxy acrylate thin polymer coated on Si microbridge. When the polymer layer absorbed methanol gas, the swelling of the polymer layer resulted in bending of the Si microbridge, so that the piezoresistive Wheatstone circuit produced an output voltage. Huang *et al*. [15] used the direct polymer patterning on substrate technique to produce a silicone-carbon black polymeric chemical vapor sensor. The sensor was sensitive to methanol, ethanol, acetone, and 2-propanol. Martinez *et al*. [16] fabricated a gas microsensor using micromachining technology. The gas sensor was used to detect methanol and carbon monoxide gases. To enhance the sensitivity of the gas microsensor, the porous tin oxide nanoparticles were prepared as the sensitive material. The structure of the gas sensor contained a MEMS microhotplate platform with interdigitated electrodes, and the sensitive material was deposited on the microhotplate platform using the micropipetting. Rahman *et al.* [17] developed a methanol sensor using micromachining technology. The sensitive material of the sensor was silver oxide doped zinc oxide nanoparticles prepared by the hydrothermal method using reducing agents in alkaline medium. The silver oxide doped zinc oxide film was deposited on microchip. The measurement showed that the sensor had a fast response to methanol in the liquid phase. Shin *et al*. [18] employed MEMS technology to make a gas microsensor with piezoelectric microcantilever. The microcantilever was composed of Pb(Zr,Ti)O_3_ (PZT) capacitors and a SiN_x_ layer. The sensitive material of the sensor was polymethylmetacrylate, which was coated on the surface of microcantilever. The sensitivity of gas sensor was 0.03 Hz/ppm for methanol vapor.

Various microactuators and microsensors developed by the CMOS process are called CMOS-MEMS technique [19,20,21,22]. Usually, micro devices fabricated by the technique require a post-CMOS process to add the functional materials [23,24] and to release the suspended structures [25,26,27]. In this work, a methanol sensor with a heater is made using the CMOS-MEMS technique. Tin dioxide doped cadmium sulfide is employed as the sensitive material of the sensor. The methanol sensor needs a post-COMS process to release the suspended structures and to coat the sensitive material. The sensor is a resistive type. A readout circuit is utilized to convert the resistance of the sensor into the output voltage.

## 2. Structure of the Methanol Sensor

**Figure 1:**
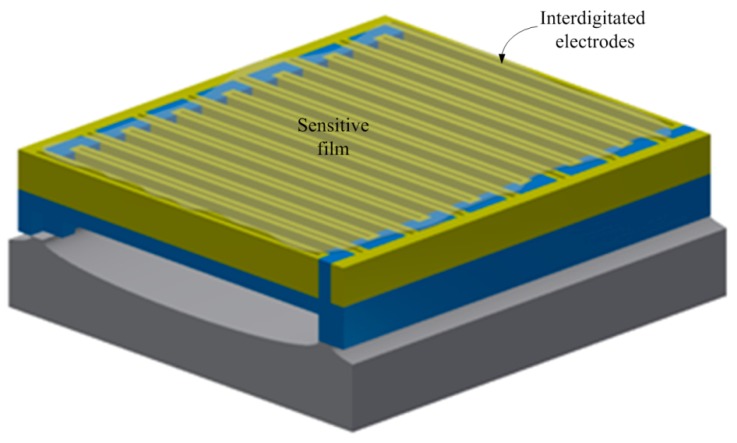
Schematic structure of the methanol sensor.

The methanol sensor chip contains interdigitated electrodes, a heater and a sensitive film. The schematic structure of the methanol sensor is shown in Figure 1. Area of the sensor chip is about 0.6 mm^2^. The tin dioxide doped cadmium sulfide is adopted as the sensitive film of the sensor, and the film is deposited on the interdigitated electrodes. The interdigitated electrodes are made of aluminum and tungsten metals. The dimensions of the interdigitated electrodes are 20 µm wide, 200 µm long and 6 µm thick, and the gap between the electrodes is 35 µm. The heater located under the interdigitated electrode is employed to generate a working temperature to the sensitive film. Material of the heater is polysilicon. The shape of the heater is designed as a winding line, and its dimensions are 15 µm, wide 2500 µm long and 0.9 µm thick. The reaction mechanism of tin dioxide sensing methanol gas can be expressed as [28]:

(1) $$\frac{1}{2}O_{2}+2e^{-}\to 2O^{-}\;$$

and

(2) $$CH_{4}O+3O^{-}\to CO_{\text{2}}+2H_{2}O+3e^{-}$$

where e^−^ represents a conduction electron and O^−^ is the oxygen ion on the surface of sensitive film. According to Equations (1) and (2), the mobility of electrons in the tin dioxide doped cadmium sulfide film increases when the film adsorbs methanol gas, so the resistance of the film decreases. Therefore, the methanol gas reacts with negative oxygen ions on the surface of the sensitive film when the film adsorbs methanol gas, resulting in the resistance of the methanol sensor decreases. Contrarily, the resistance of the methanol sensor increases as the sensitive film desorbs methanol gas.

**Figure 2:**
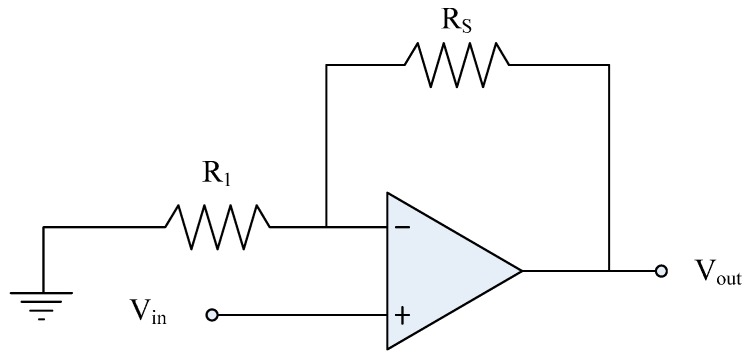
Readout circuit for the methanol sensor.

**Figure 3:**
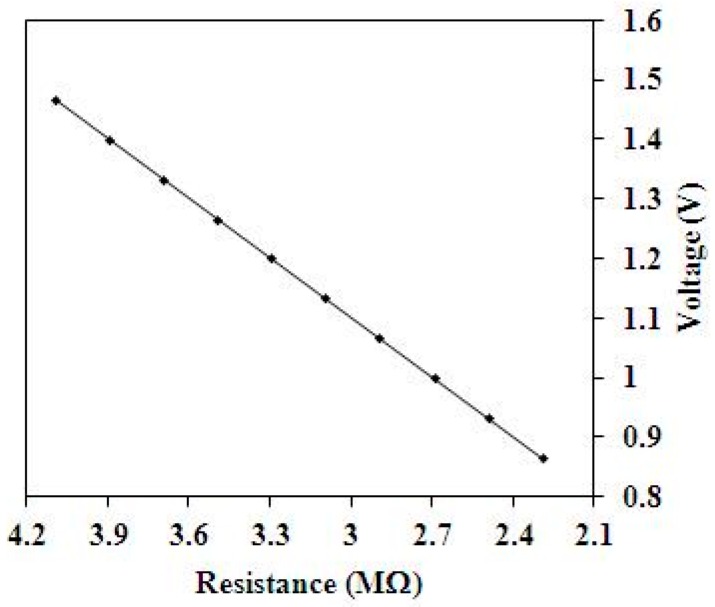
Evaluated results of the output voltage for the readout circuit.

The methanol sensor is a resistive type. The resistance of the sensor changes when it senses methanol gas. Figure 2 presents a readout circuit for the methanol sensor [29]. The readout circuit is a non-inverting amplifier, and it is adopted to convert the resistance variation of the methanol sensor into the output voltage. The circuit chip is separated with the methanol sensor chip, and they are without integration on-a-chip. The output voltage of the readout circuit can be expressed as [29]:

(3) $$V_{out}{=V}_{in}\text{(1+}\frac{R_{s}}{R_{1}}\text{)}$$

where *R_s_* denotes the resistance of the methanol sensor; *R*_1_ is the resistance of the circuit; *V_in_* is the input voltages of the circuit; and *V*_o_ is the output voltage of the circuit. The output voltage of the methanol sensor can be evaluated in accordance with Equation (3). Figure 3 depicts the evaluated results of output voltage for the circuit. In this investigation, the input voltages *V_in_* = 0.1 V and the resistance *R*_1_ = 0.3 MΩ are adopted. The resistance of the methanol sensor varies from 4.1 to 2.3 MΩ. The results show that the output voltage of the circuit decrease from 1.47 to 0.87 V as the resistance of the sensor varies from 4.1 to 2.3 MΩ.

## 3. Preparation of the Sensitive Film

Tin dioxide doped cadmium sulfide, which is prepared by sol-gel method, is adopted as the sensitive material of the methanol sensor. The tin dioxide doped cadmium sulfide was prepared as follows [30,31]: First, 6 g of tin tetrachloride was dissolved in 200 mL of deionized water with stirring for 20 min until the solution was uniformly mixed. Second, 6 mL of ammonium hydroxide was added into the tin tetrachloride solution and stirred for 30 min, and then the mixed solution was aged for 24 h. After the reaction, the resulting product was filtered, and washed with isopropyl alcohol and deionized water, which the product was tin dioxide. Third, 12 g of cadmium acetate was dissolved in 100 mL of ethanol with stirring for 30 min until the solution was uniformly mixed. Fourth, 12 g of sodium sulfide was added into the cadmium acetate solution with stirring for 30 min, followed by aging the mixed solution for 24 h. The resulting product was filtered, and washed with isopropyl alcohol and deionized water, followed by calcination at 600 °C for 2 h to form cadmium sulfide powder. Finally, cadmium sulfide powder was added into the tin dioxide with uniformly stirring at 100 °C. The product was coated on the substrate, followed by calcination at 380 °C for 3 h.

**Figure 4:**
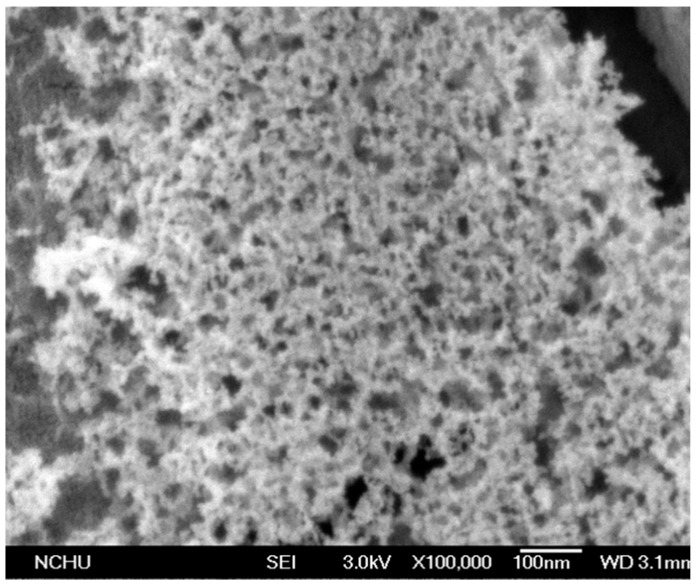
SEM image of the tin oxide doped cadmium sulfide film.

**Figure 5:**
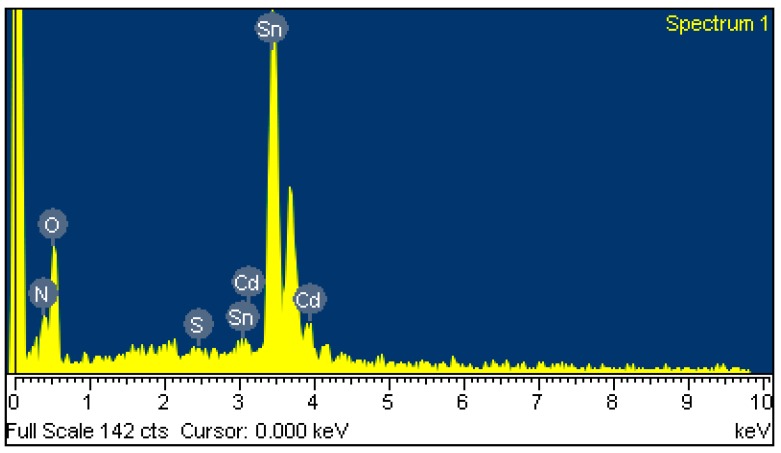
EDS analysis of tin oxide doped cadmium sulfide.

A scanning electron microscopy (SEM) [32] was used to measure the surface morphology of the tin dioxide doped cadmium sulfide film. Figure 4 shows a SEM image of the tin dioxide doped cadmium sulfide film. The film reveals a porous structure and a large surface area, which helps to enhance the sensitivity of the sensor. An energy dispersive spectrometer (EDS) was utilized to detect the composition of the tin dioxide doped cadmium sulfide film. Figure 5 shows an EDS measurement of the tin dioxide doped cadmium sulfide. The measured results showed that the composition of the film was 68.23 wt% tin, 29.22 wt% oxygen, 2.26 wt% cadmium, 0.01% sulfur and 0.28% nitrogen.

## 4. Fabrication of the Methanol Sensor

**Figure 6:**
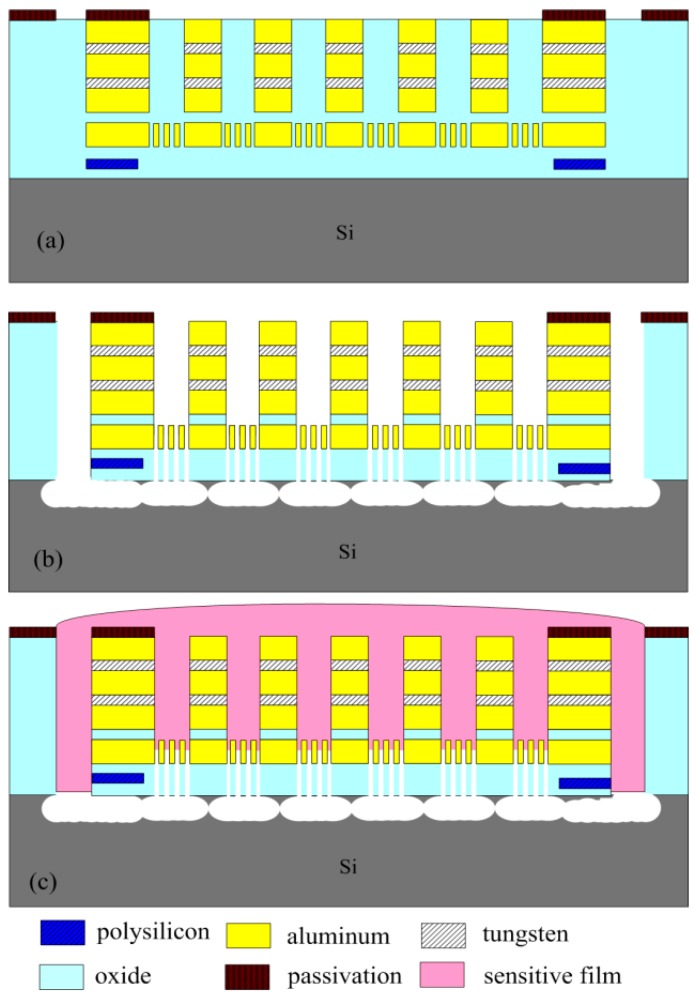
Fabrication process of the methanol sensor: (**a**) after the CMOS process; (**b**) etching the sacrificial layer; and (**c**) coating the sensitive film.

**Figure 7:**
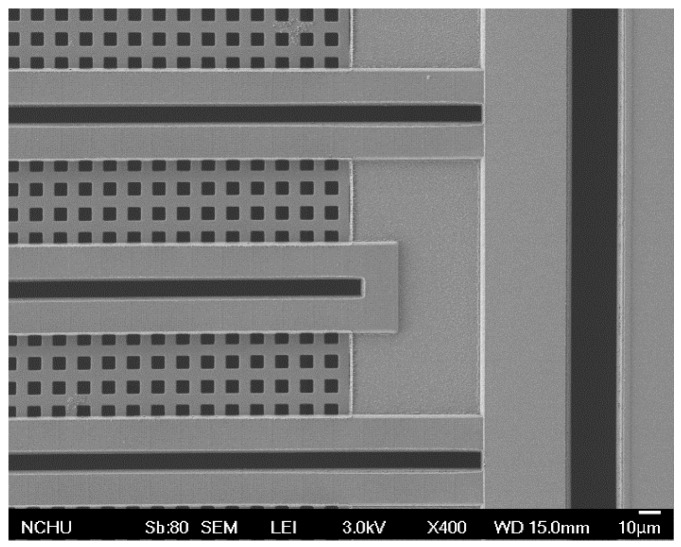
SEM image of the interdigitated electrodes after the dry etching.

**Figure 8:**
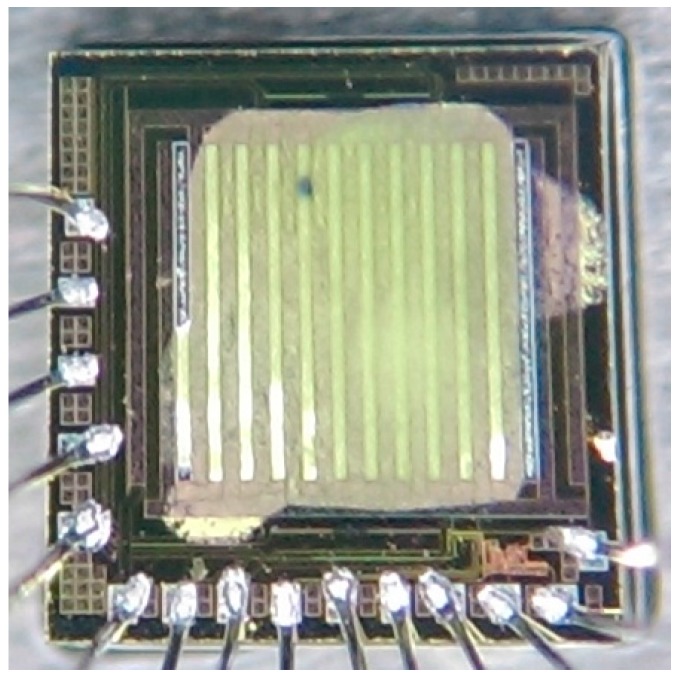
Optical image of the methanol sensor.

Figure 6 illustrates the fabrication flow of the methanol sensor chip. The commercial 0.18 µm CMOS process of Taiwan Semiconductor Manufacturing Company (TSMC, Taipei, Taiwan) was utilized to manufacture the methanol sensor chip. Figure 6a presents the cross-sectional view of the methanol sensor after completion of the CMOS process. To reduce the heat sinking of the heater, the interdigitated electrodes and the heater were designed as the suspended structures. The methanol sensor required a post-process [33] to release the suspended structures and coat the sensitive film on the interdigitated electrodes. The silicon dioxide between the interdigitated electrodes was the sacrificial layer [34]. The interdigitated electrodes were formed by a stack of aluminum and tungsten metals. Figure 6b displays that the sacrificial oxide layer is etched. An anisotropic dry etching of CHF_3_ reactive ion etching (RIE) was used to etch the oxide sacrificial layer until the silicon substrate was exposed [35]. Then, an isotropic dry etching of XeF_2_ RIE is employed to remove the silicon substrate, and to release the heater and the interdigitated electrodes. Figure 7 reveals an SEM image for a part of the interdigitated electrodes after the dry etching. Figure 6c shows that the sensitive film is coated. A precision-control micro-dropper was utilized to drop the sensitive film on the interdigitated electrodes, and then the sensitive film was calcinated at 380 °C for 3 h. Figure 8 shows an optical image of the methanol sensor after the post-process.

## 5. Results and Discussion

**Figure 9:**
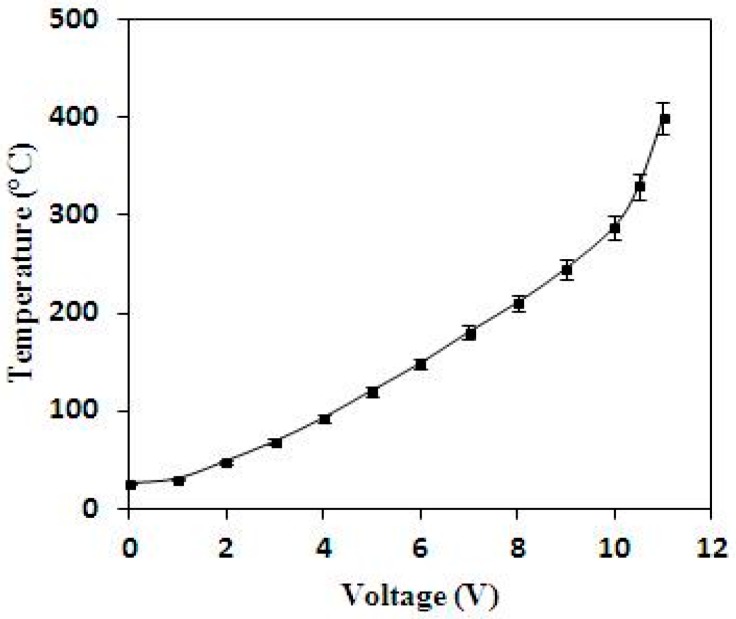
Measured results of the heater.

The performance of the heater in the methanol sensor was tested by a power supply and an infrared thermometer (NEC TVS-500EX, Nippon Avionics Co., LTD, Tokyo, Japan). The power supply supplied a voltage to the heater, and the infrared thermometer detected the temperature of the heater. Figure 9 shows the measurement results of temperature for the heater. The measured results showed that the heater produced a temperature of 360 °C when applying a voltage of 10.7 V.

**Figure 10:**
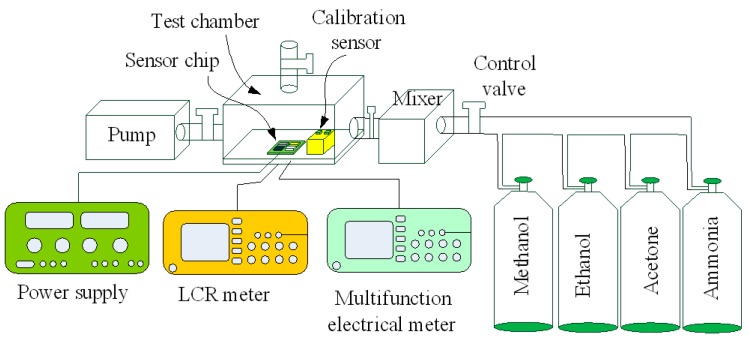
Measurement setup for the methanol sensor.

**Figure 11:**
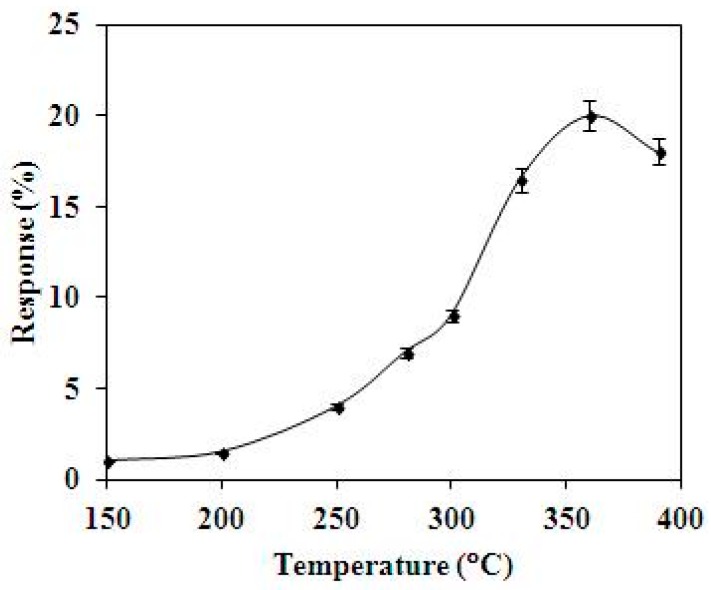
Response of the methanol sensor at 2 ppm methanol.

A test chamber, a LCR (inductance capacitance resistance) meter and a power supply were used to test the characteristic of the methanol sensor. Figure 10 shows the measurement setup for the methanol sensor. The test chamber consisted of a control valve, a pump and a calibration methanol sensor. The control valve was utilized to tune the methanol concentration in the test chamber. The pump was employed to exhaust the methanol gas in the test chamber. The calibration methanol sensor was adopted to monitor the methanol concentration in the test chamber. The sensitivity of the sensitive film in the sensor depends on temperature. To characterize the optimal working temperature of the methanol sensor, it was tested under different temperatures. The methanol sensor was set in the test chamber, and methanol gas was provided to the test chamber. The test chamber was kept at a constant concentration of 2 ppm. The heater supplied different working temperatures to the methanol sensor. The LCR meter recorded the resistance variation of the sensor. Figure 11 shows the response of the methanol sensor at 2 ppm methanol under difference temperatures. The response is defined as:

(4) $$\left|\frac{R_{s}-R_{0}}{R_{0}}\right|\times 100\%$$

where *R*_0_ represents the initial resistance of the methanol sensor and *R_s_* is the resistance variation of the methanol sensor. The measured results showed that the optimal working temperature of the methanol sensor was 360 °C.

**Figure 12:**
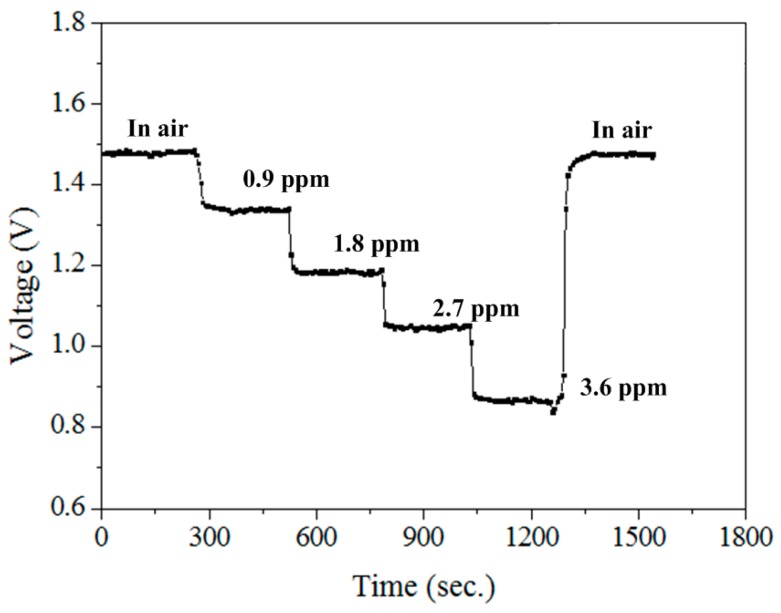
Test of the methanol sensor.

The optimal working temperature of the methanol sensor was 360 °C, as shown in Figure 11. The heater provided a working temperature of 360 °C to the methanol sensor, and the sensor was tested under different methanol concentrations. The readout circuit converted the resistance variation of the methanol sensor into the output voltage. The power supply provided a bias voltage of 5 V and an input voltage of 0.1 V to the circuit. The output voltage of the methanol was detected by the multifunction electrical meter. Figure 12 demonstrates the response of the methanol sensor at different methanol concentrations. The measured results revealed that the initial output voltage of the methanol sensor was 1.48 V in air, and the output voltage of the sensor changed to 0.88 V at 3.6 ppm methanol. Then, the sensor recovered to the initial output voltage of 1.48 V when it was in air.

**Figure 13:**
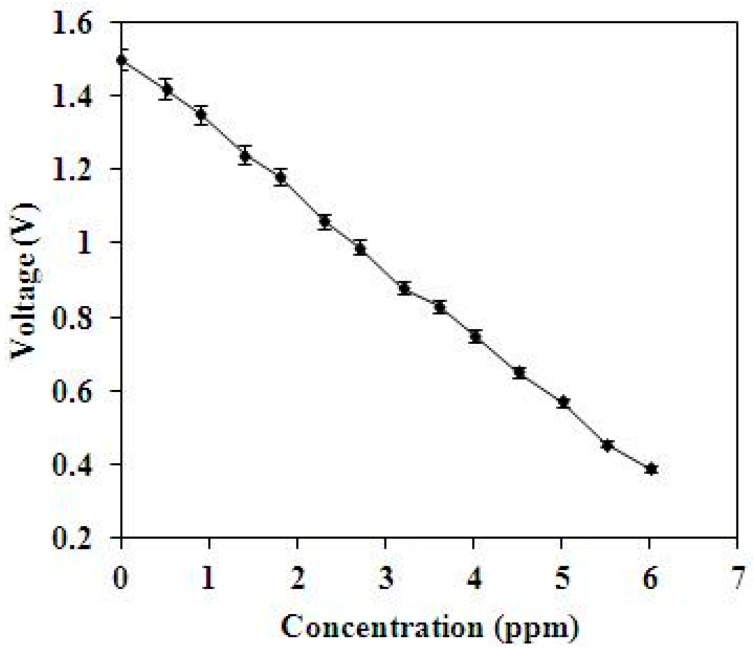
Measured output voltage of the methanol sensor.

To characterize the performance of the methanol sensor, the sensor was measured at 360 °C. Figure 13 shows the relation between the output voltage and methanol concentration for the methanol sensor. The results depicted that the output voltage of the methanol sensor changed from 1.48 to 0.39 V as the methanol concentration increased from 0 to 6 ppm. The variation of the output voltage was 1.09 mV in 0–6 ppm methanol. Therefore, the sensitivity of the methanol sensor was about 0.18 V/ppm.

**Figure 14:**
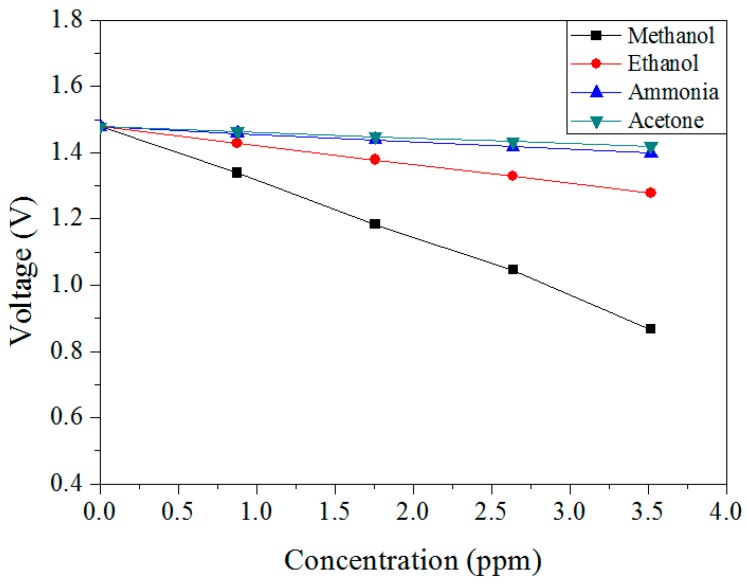
Output voltage of the sensor under different gases.

The methanol sensor was tested with different gases to characterize its selectivity. Figure 14 shows the output voltage of the sensor under methanol, ethanol, acetone and ammonia. In this measurement, the working temperature of the sensor was 360 °C. The measured results revealed that the sensor for ethanol had a sensitivity of 0.05 V/ppm, and the sensor for acetone and ammonia was non-sensitive. Therefore, the sensor had an excellent selectivity for sensing methanol.

## 6. Conclusions

A methanol sensor with a heater has been manufactured using the CMOS-MEMS technique. The sensor had the capability of detecting low methanol concentrations. The heater and interdigitated electrodes in the sensor were designed as suspended structures in order to reduce heat sinking and power consumption, so the sensor needed a post-process to release the suspended structures. The post-process used a CHF_3_ RIE to etch the sacrificial silicon dioxide layer and a XeF_2_ RIE to remove the silicon substrate, and to release the heater and interdigitated electrodes. The tin dioxide doped cadmium sulfide prepared by sol-gel method was adopted as the sensitive material of the sensor. Experiments revealed that the best working temperature of the film was 360 °C. The resistance of the methanol sensor changed as the sensitive film absorbed or desorbed methanol gas. A readout circuit was employed to convert the resistance variation of the sensor into the output voltage. The experimental results depicted that the output voltage of the methanol sensor changed from 1.48 to 0.39 V as the methanol concentration increased from 0 to 6 ppm at 360 °C. The sensitivity of the methanol sensor was about 0.18 V/ppm.
